# Large animal models for cardiac remuscularization studies: A methodological review

**DOI:** 10.3389/fcvm.2023.1011880

**Published:** 2023-03-15

**Authors:** Yuexin Yu, Seng Kong Tham, Fatin Fazrina Roslan, Bakiah Shaharuddin, Yoke Keong Yong, Zhikun Guo, Jun Jie Tan

**Affiliations:** ^1^ USM-ALPS Cardiac Research Laboratory, Advanced Medical and Dental Institute, Universiti Sains Malaysia, Penang, Malaysia; ^2^ Henan Key Laboratory of Cardiac Remodeling and Transplantation, Zhengzhou Seventh People's Hospital, China; ^3^ Henan Key Laboratory of Medical Tissue Regeneration, Xinxiang Medical University, China; ^4^ Celestialab Sdn Bhd, Kuala Lumpur, Malaysia; ^5^ Department of Human Anatomy, Faculty of Medicine and Health Sciences, Universiti Putra Malaysia, Serdang, Malaysia

**Keywords:** cardiac regeneration, large animal models, pluripotent stem cells (PSC), cardiomyocytes, cardiac remuscularization, myocardial infarction

## Abstract

Myocardial infarction is the most common cause of heart failure, one of the most fatal non-communicable diseases worldwide. The disease could potentially be treated if the dead, ischemic heart tissues are regenerated and replaced with viable and functional cardiomyocytes. Pluripotent stem cells have proven the ability to derive specific and functional cardiomyocytes in large quantities for therapy. To test the remuscularization hypothesis, the strategy to model the disease in animals must resemble the pathophysiological conditions of myocardial infarction as in humans, to enable thorough testing of the safety and efficacy of the cardiomyocyte therapy before embarking on human trials. Rigorous experiments and *in vivo* findings using large mammals are increasingly important to simulate clinical reality and increase translatability into clinical practice. Hence, this review focus on large animal models which have been used in cardiac remuscularization studies using cardiomyocytes derived from human pluripotent stem cells. The commonly used methodologies in developing the myocardial infarction model, the choice of animal species, the pre-operative antiarrhythmics prophylaxis, the choice of perioperative sedative, anaesthesia and analgesia, the immunosuppressive strategies in allowing xenotransplantation, the source of cells, number and delivery method are discussed.

## 1. Introduction

Heart failure (HF) is a disease in which the ventricular filling and/or myocardial contraction are compromised due to irreversible pathological modelling in the cardiac structure and function (1–3). Ischemic heart disease is the most common cause of HF. More than 26 million people are living with a failing heart worldwide (4), and 1 in every 8 reported deaths was due to HF (5). This prevalence is expected to increase by 46% in 2030 (6, 7). The most common cause of HF is ischemic heart disease. Myocardial ischemia as a result of the blockage in the coronary artery can affect myocardial contractility, and electrical conduction, and alters cardiac energetics (8). Pathological hypertrophic remodelling of the left ventricle, fatal cardiac tachyarrhythmia (9) followed by a cascade of secondary damage including inflammatory reaction, myocardial cell rupture and fibrotic scarring could collectively cause an abrupt decrease in left ventricular ejection fraction (LVEF) and failure. Despite technological advancement and all the current pharmacological-based treatment options, effective therapy that could prevent damaged hearts from remodelling and failure is still lacking. Heart transplantation is the only cure for end-stage heart failure, but the therapy is challenged by the lack of donor and graft rejection.

Stem cells have been the research interest and hope to remuscularize the weakening, injured myocardium and reverse cardiac remodelling. Compelling evidence has shown that pluripotent stem cells (hPSCs), such as embryonic stem cells (ESCs) or induced pluripotent stem cells (hiPSCs) offer the indefinite source of cardiomyocytes and are the only cells which can be scaled up to produce a clinical relevant number for remuscularizing injured myocardium. Studies have shown that intramyocardially injected hPSCs-cardiomyocytes engrafted, integrated synchronously with the host myocardium, regenerated the remodelled, thin myocardium and improved cardiac function. Whilst the clinical benefits of other adult stem cells such as mesenchymal stromal cells or cardiac-derived progenitor cells in cardiac regeneration are widely acknowledged (10, 11), the extent of remuscularization in the injured hearts was generally far more encouraging using hPSC-cardiomyocytes, as demonstrated in the past studies (12–15).

To establish a suitable disease model for reliable experimentation, investigators need to take into account the animal species of choice, the subject availability, the cost and the similarity/difference between the human subject as well as the methods to induce myocardial injury that produces the appropriate pathological microenvironment mimicking clinical conditions which is suitable for testing therapeutic remuscularization intervention. Table 1 summarizes the different considerations in the use of small animals (mice, rats) and large animals (pigs, dogs, monkeys) for modelling ischemic heart disease.

**Table 1:** Considerations in the Selection of Animal Category for Modelling Myocardial Infarction.

	Small animals	Large animals
** **	Mice, Rats, Guinea Pigs	Pig, Dog, Sheep, Monkey
**Life span**	Short	Relatively long
**Laboratory turnaround time**	Short	Long
**Heart Rate**	Fast (400–600 bpm in mice)	Close to humans (50–116 bpm in swine)
**Heart size**	Small	Big and close to human size
**Maintenance and research cost**	Low	Relatively high
**Heart anatomy and kinetic**	Small, rapid heartbeat	Larger, close similarity to the human heart
**LAD coronary collaterals**	Difficult to identify.	Easy to identify. Targeted occlusion at specific segments of the coronary artery can be achieved.
**MI induction method**	Mostly involved invasive thoracotomy for LAD coronary artery ligation with or without reperfusion. Other methods such as microembolism are possible but less common in cell therapy research.	Various CA occlusion methods are possible such as ameroid constrictor, coil embolism or non-invasive percutaneous balloon angioplasty inflation to induce occlusion
**Analysis of Imaging**	Expensive, high-resolution imaging equipment specialized for the small animal is needed	Imaging devices used in humans can be deployed.

## 2. Cardiac remuscularization study using large animals – the importance and rationale

An ideal disease model should mimic the pathophysiology in humans in order to more accurately and reproducibly examine any novel therapy that would successfully translate into clinical applications. In preclinical research, any new therapy would first be tested in small animal models. This is because small animals such as rodents have a short life cycle, hence requiring low maintenance cost and high availability are instrumental in producing statistically meaningful analysis within a comparatively shorter time over the use of large animals (Table 1). An early rat HF model was established by Pfeffer et al. using coronary artery ligation (16). The groundbreaking study dated back to 1979 also served as an important foundation for the development of the successfully translated drug Captopril, the angiotensin-converting enzyme that profoundly improved heart function and survival in post-myocardial infarction (MI) patients (17).

Nonetheless, the differences in heart anatomy, size, hemodynamic characteristics and responses to drugs or treatment between small animal models and humans are likely the cause of failed translation to clinical trials. These differences could also explain the rather disappointing clinical results observed in most major human stem cell therapy trials, despite the overwhelmingly positive outcome and optimism reported in laboratory small animal studies. In 2016, Zwetsloot et al. presented a systematic review and meta-analysis of preclinical studies involving cardiac stem cell treatment in MI animal models (18). They concluded that the magnitude of effects of CSC treatment in the small animal MI model was found to be greater than that of large animals. Moreover, the recent incidence of cardiac arrhythmias reported from non-human primate and pig studies following human cardiomyocyte transplantation was not previously observed in small animals possibly due to their high heart rate (13, 19, 20). Hence, small animal studies may offer important preliminary insights about the tested treatment, but a reassessment of its therapeutic efficacy must be performed on large animals whose systems are physiologically and anatomically more resembles humans prior to starting clinical trials. This is also in line with the recommendations by the transnational alliance for regenerative therapies in cardiovascular syndromes (TACTICS) international group that large mammals should be used as confirmatory studies in view of their resemblance to human disease (21).

## 3. Strategies to induce myocardial infarction in large animals

Many strategies have been introduced to create MI and HF models in large animals, with the primary objective to occlude major coronary vessels and induce ischemic injury. These include invasive thoracotomy-enabled permanent left anterior descending (LAD) coronary ligation, reversible LAD coronary artery ligation ischemic reperfusion-induced myocardial injury, coronary micro-embolism, hydraulic occluder or ameriod constrictors, or less-invasive percutaneous transluminal coronary angioplasty (PTCA) balloon occlusion-reperfusion of the coronary artery (Table 2). Nevertheless, the most common strategies employed in recent preclinical remuscularization studies using large animals were mainly the thoracotomy/permanent LAD ligation (24) or ischemic reperfusion (30) and PTCA-assisted balloon occlusion/reperfusion of LAD contrary artery (20). Unlike others, these methods allow occlusion to take place at the specific location of the LAD coronary arteries and produce predictable, consistent infarct size which is pivotal for remuscularization study. Notably, the mortality of the LAD occlusion method in large animals is considerably high as they are prone to surgical-induced trauma, high risk of bleeding and developing fatal ventricular fibrillation following MI (35).

**Table 2:** Summary of the methodologies used in in vivo cardiac remuscularization studies using large animals.

Authors, Animal Species Used and Weight	Method To Induce Myocardial Infarction	Cardiomyocyte Source, Number & Media for Cell Delivery	Time, Delivery Route and Cell Injection Method	Anesthesia and Analgesia	Immunosuppression	Prophylaxis of Infection	Reference
Nakamura K. et al. (2021) 30–40 kg castrated male Yucatan minipigs between 6 and 13 months of age	***Pre-MI Preparation*** i.v. 150 mg amiodarone and 100 mg lidocaine heparin (activated coagulation time > 250 s) ***MI-induction method*** I/R: Angioplasty balloon catheter was placed into the mid-LAD distal to the first diagonal branch artery and inflated to obstruct distal coronary perfusion for 90 min, after which the balloon was deflated to restore distal perfusion.	***Source*** Human ESCs H7 and RUES2 ***Dose*** 500 × 10^6^ hESC-CMs RPMI-1640 to achieve a target cell density for injection of ∼3 × 10^9^ cells/ml in 1.6 ml.	• 14 days post-MI • Trans-epicardial injections, or later by percutaneous trans-endocardial injections	***Anaesthesia*** i.m. butorphanol, acepromazine and ketamine. Animals were intubated, ventilated and anaesthetized using isoflurane. ***Analgesia*** subcutaneous Buprenorphine SR-La	• **Abatacept (CTLA4-Ig)** i.v. 12.5 mg/kg, on the day of transplantation, and every 2 weeks thereafter. • **Cyclosporine A** trough level was decreased to >400 ng/ml • **Methylprednisolone** reduced to 1.0 mg/kg for subjects 7–19 without histologic evidence of rejection.	Oral cephalexin Prophylactic sulfamethoxazole/trimethoprim (whenever applicable) Prophylactic valganciclovir (whenever applicable)	(22)
Zhao et al. (2021) 2-month-old Female Yorkshire pigs, 18kg	***MI-induction method*** I/R: A left thoracotomy to expose the heart through the 4th intercostal space. Occlusion was performed at the roots of the first and second diagonal coronary arteries from the LAD coronary artery for 1 h before reperfusion.	***Source*** Cardiomyocytes were generated from hiPSCs (GRiPS line) reprogrammed from human cardiac fibroblasts by using a CytoTune-iPS Reprogramming Kit ***Dose*** 3 × 10^7^ cells in phosphate-buffered saline	• Immediately after reperfusion • Intramyocardial injection onto 5 sites around the infarcted area.	***Anaesthesia*** Intubated and inhaled 2% isoflurane ***Analgesic*** Subcutaneous injections of buprenorphine SR (0.24 mg/kg) every 72 h for up to 3 days i.m. of carprofen (4 mg/kg) every 24 h for up to 2 days	NA	NA	(23)
Tan et al. (2021) Yorkshire-landrace swine (∼13 kg body weight).	***MI-induction method*** Permanent LAD coronary artery ligation: Left lateral thoracotomy and ligation of the 1st branches of LAD and left circumflex coronary arteries.	***Source*** Cardiomyocytes from PCBC hiPSC line. ***Dose*** 1.2 × 10^8^ cells in 1 ml of RPMI	• Immediately after MI induction. • Intramyocardial injection into the border and infarct zones of MI • 10 injections with each injection containing about 1.2 × 10^7^ cells. RPMI without or with Tb4-microspheres, into the border and infarct area through an epicardially applied fibrin/thrombin patch	***Anaesthesia*** i.m., 1 ml/10 kg b.w. of mixed 100 mg/ml ketamine /20 mg/ml Xylazine solution, was maintained with 2–2.5% isoflurane on a ventilator after intubation. ***Analgesia*** (Ketoprofen 5 mg/kg/day)	**Cyclosporin** (15 mg/Kg bw, from 3 days before MI surgery until euthanized	15 mg/kg Enrofloxacin, post-surgery.	(24)
Suzuki K, et al (2021) Male Gottingen minipigs (20 to 25 kg)	***MI-induction method*** The ameroid ring was attached to the proximal left anterior descending coronary artery *via* left thoracotomy.	***Source*** Cardiomyocytes were generated from hiPSCs ***Patch*** hiPSCs- derived cardiomyocytes (2.5 × 10^7^ cells/sheet) were seeded on the aligned nanofiber in a 2.5 × 2.5 cm poly-dimethylsiloxane (PDMS) frame for 2 days	• 4 weeks after MI • transplanted 4 sheets of 1 × 10^8^ CMs on nanofiber scaffold into the infarct region of the heart	***Anaesthesia*** inhalation of isoflurane	• Tacrolimus daily intake of 0.75 mg/kg per day • Mycophenolate mofetil daily intake of 500 mg/day • Corticosteroids daily intake of 20 mg/day	NA	(25)
Romagnuolo et al. (2019) Yorkshire pigs, 20-30 kg male	***Pre- MI preparation*** Pre-operative bolus of 75 mg amiodarone, 20 mg bolus lidocaine (3 mg/kg/hour infusion), and heparin (100 IU/kg iv). ***MI Induction Method*** I/R: complete occlusion of the mid LAD coronary artery for 90 min using percutaneous balloon dilation catheter, followed by reperfusion.	***Source*** Cardiomyocytes were generated using the GMP pedigree ESI-17 hESCs and HES-2 hESC line. ***Dose*** 1 × 10^9^ hESC-CMs in 3 ml PSC media PSC Media RPMI-1640 and GFR-Matrigel (∼60% v/v), supplemented with 200 nM cyclosporine A, 50 µM pinacidil.	• 3 weeks/20 days post-MI • Trans-epicardial injection • 12 injections of 250 µl cell each directly into the infarct zone using a curved 27G needle.	***Anaesthesia*** intramuscular injection of 0.05 mg/kg atropine and 33 mg/kg ketamine, followed by maintenance with 5% inhaled isoflurane. ***Analgesic*** NA	Immunosuppression began 5 days prior to cell transplantation until euthanasia at either 2- or 4 weeks post-transplantation. • Abatacept CTLA4-Ig) 12.5 mg/kg on the day of cell transplantation and every 2 weeks thereafter. • Methylprednisolone 250 mg on the day of hESC-CM transplantation followed by 125 mg/day for two weeks and then 125 mg daily maintenance thereafter); • Cyclosporine A 10-16 mg/kg PO twice per day to achieve trough concentrations of 250 µg/L, from 5 days prior to cell transplantation daily until euthanized.	NA	(13)
Kashiyama N et al. (2019) Cynomolgus macaques (6 years old, 4–6 kg)	***MI-induction method*** Permanent LAD coronary artery ligation	***Source*** Cardiomyocytes from Cynomolgus macaque iPS cell line (1123C1-G) ***Dose*** Four pieces of iPSC-cardiac sheets (3.6 × 106 cells/sheet) derived from a cynomolgus macaque	• 14 days post infarction • Epicardial implantation of iPSC-CM sheets onto the surface of the LV	NA	• Tacrolimus Continuous i.v. infusion of 0.5 mg/kg/d just before cell transplantation to maintain serum trough levels at >5–10 ng/ml until euthanized • Mycophenolate mofetil 40 mg/kg/d, oral, from 3 days before cell transplantation until euthanized. • Prednisolone 1 mg/kg/d, oral, from 3 days before cell transplantation until euthanized	NA	(26)
Ishida et al (2019) CLAWN miniature swine (weighting 18-25 kg, 6-10 months old)	***MI Induction Method*** 2.5-mm ameroid constrictor placed on the proximal LAD coronary artery Treatment for intraoperative arrhythmias Lidocaine i.v. bolus, 2 mg/kg Electrical defibrillation	***Source:*** Human iPSC (253G1, RIKEN, Japan) ***Dose*** 1 × 10^8^ cells in total, made of 10 x cell sheets using temperature-responsive poly-N-isopropylacrylamide coated surface.	• 1 month after ligation • Cell sheets were transplanted to cover the infarcted and the border regions of the affected myocardium	***Pre-emptive sedation and Anesthesia*** i.m. Ketamine (20 mg/kg), Xylazine (2 mg/kg) 2% isoflurane ***Intraoperative Analgesia*** Continuous injection of 6 mg/kg/h propofol	Peri-operative Tacrolimus (50 mg i.v.) Supplemented in food • Tacrolimus (1 mg/kg/day) • MMF (500 mg/day) • Corticosteroids (20 mg/day)	NA	(27)
Zhu K, et al. (2018) Cynomolgus monkeys (5 to 8 years old)	***MI-induction method*** I/R: A left thoracotomy and left anterior-descending coronary artery were permanently ligated distal to the first branch with a 4-0 silk suture.	***Source*** Cardiovascular progenitor cells (CVPCs) were differentiated from hESC line H9; WiCell ***Dose*** 1 × 10^7^ of hPSC-CVPCs in 1 ml DMEM/F12	• 30 min post-MI • Injections of cells with a 29-gauge syringe to 5 sites in the peri-infarct region (2 × 10^6^ cells per site).	***Pre-emptive sedation and Anesthesia*** i.m. injection of ketamine (5 mg/kg) and midazolam (0.2 mg/kg). Animals were ventilated with an animal ventilator	• Cyclosporine A orally. 30–45 mg/kg per day was adjusted to maintain serum concentrations of 100 to 250 ng/ml from 5 days before MI injury and cell administration • Methylprednisolone i.v. 500 mg, one day prior to cell administration and daily (1 mg/kg) • Simulect/Basilliximab i.v. 10 mg per dose, 2 h before cell administration and 4 days afterwards	NA	(28)
Liu et al. (2017) Macaca nemestrina monkeys, 5.2-12.6 kg	***Pre- MI preparation*** • 1 mg/kg Lidocaine bolus at a rate of 20 μg/kg/min. • Heparin i.v. to maintain activated clotting time of 250-350 s ***MI Induction Method*** IR: A coronary guide wire and angioplasty balloon (Apex 2 × 8 mm PTCA dilatation catheter) induced occlusion at mid-LAD for 180 min Animals were left for 14 days after myocardial infarction before treatment with cells.	***Source*** Cardiomyocytes were differentiated from RUES2 and H7 human embryonic stem cells. ***Dose*** ∼750 × 10^6^ CM in 1.5 ml PSC media ***PrSC Media:*** RPMI-1640 10 μM ZVAD-FMK/Caspase Inhibitor 50 nM TAT-BH4/BCL-XL 200 nM Cyclosporine A 50 μM Pinacidil and 100 ng/ml IGF-1	• 14 days post-MI • Intramyocardial injection • 15 cell injection from epicardium into infarct and peri infarct region, each at 100 μl in volume • (50 million cells per injection)	***Anaesthesia*** sedation using ketamine and propofol. Endotracheal Intubated and ventilated using sevoflurane or isoflurane ***Analgesia*** Buprenorphine	• Cyclosporine i.v. to maintain serum levels of 200–250 μg/L 5 days before cell injection and maintained until macaques were euthanized. • Methylprednisolone i.v. 30 mg/kg a day before cell delivery, 6 mg/kg for the subsequent 2 days. 3 mg/kg thereafter until the monkeys were euthanized. • Abatacept (CTLA4-Ig) 12.5 mg/kg subcutaneously a day before cell delivery and every 2 weeks until monkeys were euthanized.	• Ceftazidime, • Cefazolin, • Vancomycin, • Gentamycin, Fluconazole, • Acyclovir (* doses not mentioned)	(12)
Shiba Y. et al. (2016) 4–5-year-old female Cynomolgus monkeys	***Pre- MI preparation***1 mg kg−1 lidocaine and 1000 U heparin, i.v., heparin every h until reperfusion. ***MI Induction Method*** I/R: MI model was produced by 3 h mid LAD ligation followed by reperfusion using polyethene tubing 2 weeks before transplantation	**Source** male MHC-homozygous cynomolgus monkey iPSCs ***Dose:*** iPSC-CMs (4 × 10^8^) ***Media:*** CMs suspended in PrSC (29) or PSC vehicle ***Pro-survival cocktails*** • Not listed. Cited Laflamme et al. (2007) (29)	• Two weeks post-MI • Intramyocardial injection into the infarct zone and the border zone • 10 injections of 100 μl each using a 29-gauge injection needle.	***Anaesthesia*** i.m. injection of ketamine and xylazine, intubated with a 4-mm tracheal tube and ventilated with 1.5% isoflurane. ***Analgesia*** Buprenorphine	• Methylprednisolone 10 mg kg^−1^ day^−1^ (daily intra-muscular injection) from the day before transplantation for 3 days and at 1 mg kg^−1^ day^−1^ thereafter. • Tacrolimus 0.1 mg kg^−1^ day^−1^, daily intra-muscular injection 2 days before transplantation.	NA	(30)
Chong et al (2014) Macaca nemestrina, 8.6–12.3 kg	***Pre- MI preparation*** • Daily 100 mg amiodarone (5 days before MI to 10 days after MI) • i.v. lidocaine bolus 1 mg/kg and infusion 20 mcg/kg/min. • i.v. Heparin to maintain activated clotting times of 250–350 s ***MI Induction Method*** I/R: Percutaneous balloon Occlusion at mid-LAD coronary artery using a coronary guide wire and angioplasty balloon (Apex 2 × 8 mm PTCA dilatation catheter) inflated for 90 min	**Source** Cardiomyocytes were differentiated from RUES2 and H7 human embryonic stem cells ***Dose*** 1 × 10^9^ CMs in 1.5 ml PSC Media ***PrSC Media:*** RPMI-1640 50% (v/v) GFR Matrigel 50 nM BCL-XL BH4 200 nM Cyclosporine A 50 mM Pinacidil 100 ng/ml IGF-1	• 14 days post-MI • Intramyocardial • 15 injections each of 100 μl volume into the infarct region and adjacent border-zones. • 3 injections through the cells were injected through a single epicardial puncture, but with 3 changing trajectories of the needle for each. The needle tip wound was closed up with the mattress suture.	***Anaesthesia*** Ketamine and propofol, intubated and ventilated using sevoflurane to maintain anaesthesia ***Analgesia*** Fentanyl and buprenorphine	• Methylprednisolone i.v. 500 mg on the day of hESC-CM delivery maintenance doses of 0.1–1.5 mg/kg until sacrifice, • Cyclosporine serum trough levels of 200–250 μg/l from 5 days prior to hESC-CM delivery until sacrifice and • Abatacept (CTLA4 -Ig) 12.5 mg/kg on the day prior to hESC-CM and every 2 weeks thereafter.	Broad-spectrum antibiotics and anti-fungal agents. (Types and doses not mentioned)	(20)
Ye L. et al. (2014) Female Yorkshire swine (∼13 kg, 45 days of age)	***MI-induction method*** I/R: ligation of the D1 branch of the LAD and the M1 branch of the left circumflex coronary arteries with 4.0 polypropylene sutures, occluded for 60 min; and reperfused for 15 min.	***Source*** HiPSC lines used for cardiomyocytes, ECs and SMCs were from DriPS16 and GRiPS lines ***Dose*** 2 × 10^6^ hiPSC-CMs, 2 × 10^6^ hiPSC-ECs, and 2 × 10^6^ hiPSC-SMCs (6 × 10^6^ cells total) ***Patch*** Day-10 hiPSCs- derived cardiomyocyte cell sheet containing 10 × 10^6^ cells suspended in 25 mg/ml fibrinogen, covered area of myocardial infarction.	• 15 min after reperfusion • Cell: Intramyocardial injection • Patch: Epicardial implantation	***Anaesthesia*** NA, cited (31, 32) below Pentobarbital (30 mg/kg followed by a 4 mg/kg/hr IV), intubated and ventilated with oxygen.	• Cyclosporine A 15 mg/kg per day with food from 3 days before MI Induction until euthanized.	NA	(33)
Kawamura M, et al. (2012) Female minipigs (20 to 25 kg)	***MI-induction method*** Ameroid constrictors were placed around the left anterior descending coronary artery	***Source*** Cardiomyocytes were generated from hiPSCs line 201B7 ***Patch*** Day∼27 hiPSCs- derived cardiomyocytes scaffold-free sheet was created using thermoresponsive dishes and transplanted over the MI site	• 4 weeks after MI • Cell sheets of 1-2 × 10^7^ human iPSC-derived cardiomyocytes	Anesthesia Ketamine hydrochloride (20 mg/kg) and xylazine (2 mg/kg) General anesthesia was achieved by propofol infusion (6 mg/kg/h) and vecuronium bromide (0.05 mg/kg/h)	Tacrolimus (0.6 mg/kg), daily starting 5 days prior to transplantation until euthanized.	NA	(34)

MI, myocardial infarction; hiPSC, human induced-pluripotent stem cells; PrSC, pro-survival cocktails; I/R, ischemic-reperfusion injury; i.v. intraveneous injection; i.m., intramuscular injection; LAD, left anterior descending; ZVAD, benzyloxycarbonyl-Val-Ala-Asp(O-methyl)-fluoromethyl ketone; CTLA-Ig cytotoxic T-lymphocyte-associated protein 4-immunoglobulin; bw, body weight. PTCA, percutaneous transluminal coronary angioplasty.

For assessing the efficiency and efficacy of cardiomyocyte therapy on cardiac remuscularization, the animal model must develop clear infarction to create the need for cellular reconstitution. Methods such as coronary microembolism, pacing-induced tachycardia and toxic injury can induce dilated cardiomyopathy even without the presence of clear infarcts, making them less common methods used for the cardiac remuscularization-related study (36).

### 3.1. Irreversible occlusion of left anterior descending coronary artery

Among the most commonly used methods for modelling MI in large animals is by surgically ligating the LAD coronary artery (37), with or without reperfusion (35). This method requires invasive thoracotomy and complex surgical procedures to minimize unnecessary tissue injury, infection and animal suffering that allow good post-operative recovery. The advantage of this method is the convenience of getting a direct visual of the heart anatomy to identify and choose the site of ligation along the LAD coronary artery since the ligation site determines the resultant infarct size, as well as the mortality rate of the animal (38, 39). In Tan et al. (2021) study, they performed a permanent LAD coronary ligation without reperfusion on Yorkshire-landrace swine (∼13 kg) heart, with the ligature placed at the first branches of LAD and left coronary circumflex (24). This led to an ejection fraction of ∼40% with a scar size of 15% in the swine MI model. Some studies presented rather arbitrary and confusing descriptions in their methodology especially the choice of ligation site, which may explain the inconsistencies that complicate the inter-study analysis (40). In Kashiyama et al. study, they reported using permanent LAD coronary artery ligation in cynomolgus macaques (6 years, 4–6 kg) but without specifying the exact segment of LAD coronary where the ligature was placed (26). In their study, the ejection fraction was found to decrease by 30% in the control macaques.

Noteworthy that in MI models, there are differences in the coronary collateral perfusion network in the animal species of choice which can significantly affect the resultant infarct size and the extent of the ischemic bed. In swine and non-human primates, the coronary arterial architecture with limited collateral vessels is similar to that of humans (41, 42). Therefore, a ligation at a specific coronary segment can produce consistent and predictable infarct size. The only setback in using the swine model is the rapid changes in body weight from juveniles and this may complicate long-term analysis as this alters the baseline of swine cardiac physiology (43). Canine, on the other hand, has an extensive collateral coronary network (44). Placement of the ligature at the similar coronary artery segment may not produce the intended, consistent infarct size (45), because the infarct size decreases as the collateral flow in the canine heart increases (46).

### 3.2. Gradual LAD coronary occlusion using ameriod constrictor

Ameroid constrictor was first introduced in 1957 (47), consisting of an inner hygroscopic casein ring that, upon fluid absorption, gradually expands against a fixed metal sheath and occludes the vessel where it is placed (48). This method induces chronic progression of coronary artery disease and develops chronic myocardial ischemia in large animals, especially in pigs. LAD occlusion using an ameroid constrictor demonstrated a steady decline in cardiac function from 8 to 12 weeks, with a 50% reduction in the ejection fraction (49), inducing chronic HF in the mini swine model with high reproducibility (50). Ameroid constrictor comes in various sizes to fit the diameter of the vessel of interest which is to be constricted. A study has shown that different device size (tested 2.25, 2.50 and 2.75 mm) has minimal effects on the resultant coronary flow and ischemic area following the constriction in pigs (51). This may not be the case in the canine model as the collateral vessels gradually develop and reduce the regional ischemia and infarct size (51). The modelling of chronic coronary stenosis using ameroid constrictors in large animals is generally less popular due to the laborious animal care and maintenance cost underlying the study.

### 3.3. Transient LAD occlusion to induce ischemic/reperfusion injury

In clinics, rapid recovery of coronary blood flow through balloon angioplasty is one of the treatment strategies for acute MI. Ischemic/reperfusion (I/R) injury is a paradoxical phenomenon where cellular injury results from the rapid restoration of blood flow after prolonged oxygen deprivation. This is due to the abrupt surge in oxygen level, which increases the production of free radicals and calcium influx that causes mitochondrial damage (52, 53). This is followed by an accelerated inflammatory response which deteriorates cardiac functions and causes irreversible myocardial cell death (54). To model this pathological process in animals, temporary obstruction of LAD flow could be achieved by ligating the LAD coronary artery using a silk suture with the ligature tied against a piece of polyethene tubing parallel to the artery (55). Releasing the polyethene tube would then allow reperfusion and introduce reoxygenation injury. Alternatively, cardiac ischemia-reperfusion injury can also be achieved using a minimally invasive intervention without thoracotomy, by using an inflatable intracoronary balloon catheter (12, 13, 22). Although this method omits thoracotomy and reduces invasive tissue trauma, a specialized skillset and expensive facility are also required in performing the procedure under fluoroscopy.

In the I/R injury model, the length of the induced ischemic time (or the coronary artery occlusion time) also affects the outcome of the infarct size, in addition to the location of LAD coronary artery blockage as previously mentioned. In a recent study by Silvis et al. (2021), they examined the effects of different coronary artery balloon occlusion times (60, 75 and 90 min) and the reperfusion time on the myocardial infarct size in pigs (56). They showed a positive correlation between ischemic time and infarct size determined by the area at risk. However, a longer reperfusion time after the occlusion of 75 min, examined after 1, 3 and 7 days, did not affect or exacerbate the infarct size further. In short, the occlusion time of 30–180 min in duration has proven to cause significant ischemic-induced infarction in a large animal (57, 58). In most recent studies involving cardiac remuscularization study using human cardiomyocytes, the occlusion time used in establishing the I/R MI model were 60 min (swine) (33), 90 min (swine or non-human primates) (13, 22) and 180 min (non-human primates) (30).

### 3.4. Pre-MI induction preparation to reduce the incidence of fatal arrhythmias

The incidence of arrhythmias is common in MI models, especially the two most commonly used large animal species swine (59) and non-human primates. In the anaesthetized MI porcine model, fatal arrhythmia following coronary occlusion was found to be almost inevitable (38). Approximately 15%–40% of MI models in pigs died of ventricular tachycardias (VT) and ventricular fibrillation (VF) minutes after coronary occlusion (59, 60). Some studies eliminated VF upon onset of MI in pigs by defibrillation at 200 J (56, 61).

#### 3.4.1. Amiodarone

Amiodarone is a class III antiarrhythmic drug and non-competitive beta-blocker agent indicated to treat cardiac dysrhythmias and it has been used as a prophylaxis means to prevent VF during the early onset of MI (62), and to treat VF post-AMI. The study has also shown that amiodarone, in combination with lidocaine the anaesthetics and class 1b antiarrhythmic agent (63, 64), reduced the incidence of fatal arrhythmia in the ovine MI model (65). This combination of prophylaxis regimen has also been adopted by Murry's laboratory in their macaque MI study using human embryonic stem cells-derived cardiomyocytes in 2014 (20). They administered 100 mg amiodarone daily for 5 days prior to MI induction to 10 days after MI induction through the oral route, in addition to using lidocaine bolus at 1 mg/kg and by infusion at 20 *μ*g kg^−1^ min^−1^. The same laboratory removed amiodarone from their standard of prophylaxis care in their recent report in 2018 but used it in one animal with refractory VF/VT (bolus intravenous infusion) at 85 min of ischemia and given again in combination with lidocaine (75 mg) and dopamine (10 *μ*g kg^−1^ min^−1^) for 24 h after reperfusion due to sinus tachycardia (12). However, they also acknowledged the omission of the amiodarone from their study accounted for a higher incidence of arrhythmias pre-cell injection.

#### 3.4.2. Heparin

Heparin is an anticoagulant with heterogenous size and activity (66).– The primary mechanism of action of heparin is to bind and enhance the activity of antithrombin III and is required for performing percutaneous coronary intervention procedures during I/R modelling in large animals similar to the clinical practice, in order to reduce thrombosis (67, 68). Some studies also infused heparin before introducing I/R injury even in thoracotomy-open chest coronary artery ligation surgery when PCI was not used (30), to maintain a high activated clotting time (∼250–350 s) (69), which were found to inversely correlated with the likelihood of abrupt vessel closure (70, 71).

## 4. Choice of sedative, anesthesia and analgesia

The surgical procedures required to perform *in vivo* cardiac remuscularization studies are mostly invasive, some of which require a thoracotomy, percutaneous coronary intervention, and for enabling direct intramyocardial cell injection. Handling large animals to prepare them for these procedures is challenging and inhumane without proper and effective perioperative sedation, general anaesthesia and analgesia. The outcome of infarction in swine was also reported to be affected by the choice of anaesthesia and breed (72). Employing a suitable anaesthetic strategy during MI modelling in the large animal is important for achieving adequate anaesthesia during the induction of the intended pathophysiological changes, as well as securing stable post-surgery hemodynamic and recovery, and reducing the risk of anaesthesia-related mortality due to malignant hyperthermia or complications following long anaesthesia (73). Here, the choice of pre-emptive sedatives, anaesthesia and analgesia used in large animal studies in Table 2 are discussed.

### 4.1. Pre-emptive sedation and analgesia

Large animals, such as porcine, canine or nonhuman primates require sufficient restraint for any interventions without jeopardizing the safety of the handlers. Ketamine is a widely used intramuscular administered, non-competitive N-Methyl-D-aspartate (NMDA) receptor antagonist and dissociative anaesthetic of which the state of anaesthesia is cataleptic with intense amnesia, analgesia and hypertonus (74). It is a Class III controlled substance governed by the Drug Enforcement Agency (DEA) in the United States, as well as regulated by the local authority of many countries like Malaysia.

Ketamine is more widely used for less painful interventions but offers good restraint in large animals e.g., blood collection. It is a known irritant upon intramuscular injection due to its acidity and has a high risk of developing neuronal damage and loss of sensation in rhesus macaques (75) or muscle damage in marmosets (76). It is also a direct negative inotrope and has the ability to inhibit neuronal and extra-neuronal catecholamine uptake (77). Ketamine provides fast-acting and rapid-onset sedation (78) due to its high hydrosoluble characteristic that allows a rapid increase in bioavailability in the central nervous system (79).

Most procedural sedation and pre-emptive analgesic strategy used in the studies summarized in Table 2 included ketamine prior to the induction of any inhalational anaesthetic agent. However, none of them administered ketamine as monotherapy. Instead, ketamine was combined with other drugs with analgesic effects such as xylazine, midazolam, atropine or propofol. This is because gas anaesthesia alone provides no pain control and can be stressful to the subject upon its withdrawal and recovery. Combinations with xylazine the *α*2-agonist or other NMDA-antagonist such as diazepam or midazolam can achieve good sedation and excellent analgesia. The combinations also showed to prevent seizures and promote muscle relaxation (80). One of the advantages of using both combo ketamine/xylazine and ketamine/midazolam could abrogate the swallow reflex during endotracheal intubation (81).

Ketamine can cause hypersalivation in large animals and humans (82). This can be addressed by co-administering anticholinergics atropine (82). Furthermore, other combination such as ketamine/propofol, collectively called “Ketofol”, is used for short procedural sedation (12, 20). Propofol is a sedative-hypnotic agent with good sedation and muscle relaxation effects which could address the observed ketamine-induced muscle spasm in rhesus macaques (83). Ketofol has also been found to reduce adverse respiratory events as compared to propofol treatment alone (81), as well as demonstrated neuroprotective and anti-inflammatory responses in mice with toxic status epilepticus (84). However, the combination was also associated with a high incidence of tachycardia (85, 86).

A ketamine/acepromazine/butorphanol procedural analgesia mix was used by Nakamura et al. (2021) in minipigs. Acepromazine is a phenothiazine tranquillizer of which sedation is achieved *via* inhibiting alpha-adrenergic, dopamine receptors in the central nervous system. It provides only mild to moderate sedation in pigs and requires a normal liver function to be metabolised and excreted through the kidneys. Acepromazine is not by itself an analgesic but it can enhance the effects of analgesic opioids, and in this instance, butorphanol (87). It also prolonged the anaesthetic effects of ketamine and reduced the proportionate dosage of ketamine (88).

### 4.2. Inhalation anaesthetic

The two most common inhalational anaesthetic agents used in the recent cardiac remuscularization studies in large animals are isoflurane and sevoflurane (Table 2). Isoflurane is a widely chosen inhalational anaesthesia for experimental interventions. However, it is also known for its dose-dependent depression of cardiac performance. This can be resolved by adding nitric oxide to isoflurane anaesthesia to reduce the depressant effects on the heart. Studies have also shown that isoflurane neither contributes to coronary steal at clinically-meaningful concentrations (89) nor causes myocardial ischemia in dogs (90). In fact, isoflurane has been demonstrated to have protective effects against myocardial I/R injury (91). Sevoflurane shares similar characteristics to isoflurane, but it exerts dose-dependent depression of the cardiac functions such as stroke volume, cardiac output or left ventricular contractility without affecting the heart rate (92). Noteworthy, sevoflurane was found to have a 30% lower incidence of VF than isoflurane, as well as comparatively greater hemodynamic stability and lower mortality in a porcine I/R study.

### 4.3. Post-operation analgesia

Buprenorphine and fentanyl are the two opioid analgesics of choice in large animal studies involving invasive surgery. Buprenorphine is a µ (mu) receptor partial agonist but the binding is strong and hence the effect is long-lasting and able to displace other short-acting pure *μ* agonists (93). It is also a weak *κ* (kappa) receptor antagonist, making it a weak inducer of opiate effects. Fentanyl, on the other hand, is short-acting, but a more potent, pure µ-receptor agonist. Both opioids have little effect on the heart (94, 95), but buprenorphine is more favourable than fentanyl because it causes less respiratory depression at high doses with a ceiling effect (96). Whereas fentanyl demonstrated dose-dependent respiratory depression at high doses.

Ketoprofen and carprofen are non-steroidal anti-inflammatory drugs (NSAIDs) drug with analgesic effects and have lower toxicity profiles in animals than other NSAIDs. Both are propionic acid derivatives acting through the inhibition of cyclooxygenase and impedes prostaglandins biosynthesis. These NSAIDs were found effective in postoperative pain relief in dogs (93) but have reported the risk of gastrointestinal complications including stomach ulcers (97). Ketoprofen is also known to affect platelet aggregation and care should be taken in case of gastrointestinal bleeding (98).

## 5. Cardiomyocyte source, route of delivery and cell number

Successful remuscularization of an injured heart requires promising regeneration of the dead myocardial tissue in order to restore the myocardial muscle density and contractile strength. Many cell candidates have been tested in laboratories or clinics (10), but this review will only discuss the past animal studies that used human cardiomyocytes derived from hiPSCs. Methods and the efficiency of deriving functional cardiomyocytes from human PSCs have been improved substantially, either by using growth factors, small molecules, or combinations of both at a defined culture time (99).

A myriad of methods has been introduced to administer cells into the heart, with an aim to maximize cell homing, retention, engraftment and subsequent survival and function. Previously, cell administration *via* the systemic intracoronary route in cynomolgus monkeys has proven inefficient with a high incidence of embolism and poor graft survival (100). Local intramyocardial route, however, is the most favourable method to deliver cardiomyocytes, either *via* trans-endocardial or trans-epicardial injection. The trans-endocardial route is considerably less invasive than other local injection methods as it can be achieved *via* the percutaneous catheter. The unique challenge in implanting cells into the myocardium is the characteristic of the heart being a constantly contracting organ, which creates the mechanical force that squeezes the injected cells out through the needle track (the “washout” effect) or the broken blood vessels because of direct injection (101, 102).

To achieve high cell retention, epicardial implantation of a cardiac patch may be considered. The only shortcoming of this delivery method is the inevitable, invasive thoracotomy required for the implantation. Recently, a minimally invasive intrapericardial cell injection was proposed (102). The authors performed the procedure through two small incisions (one for insertion of a camera probe and another for a needle with exosomes in hyaluronic acid hydrogel) on the pig chest wall and showed minimal inflammation. They also tested the delivery method using hiPSC-derived cardiac progenitor cells in decellularized porcine heart matrix hydrogel and demonstrated promising cell engraftment on the epicardial surface, minimal immune response as well as the evidence of *in vivo* cardiomyocytes differentiation of the injected cardiac progenitors. However, this reported benefit was only tested and observed in rat hearts.

Studies have shown that the number of transplanted cardiomyocytes determines the degree of remuscularization in the injured heart (103). The range of cardiomyocyte numbers which were tested in the∼40 g macaque infarcted hearts was between 400 and 1,000 million human induced pluripotent stem cells-derived cardiomyocytes, through intramyocardial injection (12, 20, 30). Assuming the average monkey body weight in those studies was 9 kg, the dose for every kg body weight in a human would be 44–111 million cells or 3–7 billion cells in an adult human with an average body weight of 70 kg. However, these numbers remain inconclusive, as a question was raised about the clinical relevance in proportion to the size of a human heart of which the left ventricle contains only∼5 billion cardiomyocytes (104).

Another common approach in preclinical CM transplantation studies in addition to intramyocardial injection is by epicardial implantation of engineered heart tissue. A recent study by Querdel et al. (2021) showed that EHT made of high cardiomyocyte dose (1.5 × 2.5 cm, 12 × 10^6^ cardiomyocytes) improved heart function in guinea pigs, with *in situ* time-dependent cardiomyocyte proliferation within the implanted EHT (103). The authors also claimed to have successfully upscaled to generate a 7 × 5 cm human-relevant-sized EHT with 450 million cells for clinical use. In a MI study using Gottingen minipigs (weight 20–25 kg each), Suzuki et al. (2021) transplanted four large, 2.5 × 2.5 cm cardiac tissue made with 2.5 million cardiomyocytes on the aligned nanofibers to the infarcted myocardium (1 billion cells in total, 50 million kg^−1^ for a 20 kg minipig) (25). They concluded the treatment improved cardiac function and angiogenesis with antifibrotic effects but low engraftment, possibly due to immune rejection.

## 6. Strategies to overcome xenogenic cell immune rejection and immunosuppression

In most cases, the established MI animal models used for testing the regenerative capability of any cell candidate were from xenogenic sources, e.g., human cardiomyocytes to swine or macaques' hearts. One of the key determinants of successful clinical use of cardiomyocyte therapy is dictated by the degree of engraftment and survival of the transplanted xenogenic cells and this outcome is affected by the immunologic responses of the host recipient upon transplantation. Most of the preclinical cell therapy experimentation involves xenotransplantation (12). Transplantation of non-autologous cells can result in immune reactions that are primarily caused by acute cellular rejection, mainly because of the T cell alloantigen recognition of the major histocompatibility complex (MHC) (105). Some allogenic cell candidates may have the ability to evade immunorecognition and avoid graft rejection, like the mesenchymal stromal cells (MSCs). These cells are known to be immunomodulatory-privileged and can effectively modulate the immune system by inhibiting T cell proliferation or maturation after transplantation (106). This suggests that MSC transplantation might not need immunosuppression even if the cells are of an allogenic source (107). However, such privilege was found to be withheld when the cells were differentiated (108).

The very original concept of the creation of human iPSCs was the possibility to derive them from autologous sources, and offering cell therapy with the patient's own cells for transplant would resolve the problem with immunorejection (109, 110). However, the high cost underlying each iPSC line generation and the significantly longer time (months) required for differentiation and up-scaling may not be feasible for some clinical conditions which in need of immediate treatment. Hence, getting a universal human iPSC line that could serve “off-the-shelves” would make cell therapy more readily available for the use of broader patients.

The idea of cryobanking human induced pluripotent stem cells (iPSCs) generated from HLA homologous donors matching for human leukocyte antigen (HLA)-A, HLA-B, and HLA-DR alleles has been first advocated in the United Kingdom (111). Instead of using autologous cells, matching the compatibility of the allogeneic iPSCs based on these three most notorious triggers of immune rejection would turn the cell lines transplantable for a larger patient population with good graft survival (111, 112). However, this approach may only apply to countries in which the population has low diversity in HLA haplotypes (113). Moreover, the conflicting finding was also observed in iPSC-derived cardiomyocytes which revealed the need for immunosuppressants despite the reduced immune reactivity due to MHC matching (114).

In 2019, Schrepfer's laboratory revealed that generating allogeneic human iPSCs with hypo-immunogenicity is in fact possible (115). These allogeneic human iPSCs inactivated their major histocompatibility complex MHC class I and class II genes (*B2M* and *CIITA*, respectively), as well as upregulated the non-MHC ligand CD47 to silence innate immunity. They tested the hypoimmunogenic human iPSCs in allogenic humanized NSG-SGM3 mice and showed the successful formation of teratoma. The iPSC differentiated derivatives, endothelial and cardiomyocytes from the same hypoimmunogenic line also showed similar survival in the mice up to 50 days, confirming engineering process did not compromise the iPSC function and its hypo-immunogenicity. Cowen's laboratory also suggested the removal of *CIITA* in human PSCs but they proposed selective deletion of HLA-A/-B/-C instead of B2M to preserve the expression of HLA-E and HLA-G, the HLA class Ib molecules that retain the tolerance to natural killer (NK) cells (116). They also introduced the expression of PD-L1 (T cell checkpoint inhibitor) and HLA-G in addition to CD47. In their findings, these modifications were able to be protected from the immunosurveillance of T cells, NK cells and macrophages.

### 6.1. Drug-induced immunosuppression

While awaiting to materialize the use of hypoimmunogenic hiPSC lines in clinics, an immunosuppression regimen is needed for any allogenic cell transplantation in the current *in vivo* large animal studies or in human studies. Immunosuppressive agents are used either alone or in combinations to eliminate the effect caused by host immune rejection following cell transplantation. Calcineurin inhibitors and glucocorticoids are the two common types of immunosuppressive drugs used in allogeneic cell transplantation studies in large animals and using the two in combinations is a more preferred strategy as it yielded superior effects than using a single, individual drug approach.

#### 6.1.1. Calcineurin inhibitor

Calcineurin is calcium and calmodulin-dependent serine/threonine protein phosphatase which activates T cells (117). Calcineurin inhibitor is commonly seen as the choice of immunosuppressive agent in many large animal preclinical studies of cardiac regenerative therapy, such as cyclosporine A (CsA) or tacrolimus (Table 2, Immunosuppression). CsA binds to cyclophilin while tacrolimus binds to immunophilin FK506 binding protein 12 (FKBP12) in the cytoplasm. Both complexes prevent the downstream calcineurin-calmodulin complex-mediated dephosphorylation of nuclear factor of activated T-cells (NFAT) and upregulation of interleukin-2 (IL-2), the key cytokine which activates T cell proliferation. Some studies supported that CsA can enhance the immunosuppressive capability of MSCs, but the evidence is only limited to *in vitro* observations (118, 119). A study was performed to examine the serum level for animal safety after CsA administration (15 mg^−1^ day^−1^, twice daily *via* oral route) in the I/R pig model (weight ∼33 kg) (120). All readings of the serum levels were found within the reference value, suggesting that the dose is safe to keep CsA serum concentration at 82%. This CsA dosage is still adopted in many recent pig MI studies (24, 33). In non-human primates, CsA was given to maintain a serum trough level of 200–250 μg/L (12, 20). Tacrolimus is also used to replace CsA in case of recurrent rejection (121) and has fewer side effects that are seen in CsA like hypertrichosis and gingival hyperplasia (121).

#### 6.1.2. Steroid

Glucocorticoids are steroid hormones (prednisone and methylprednisolone) that modulate the gene expression of T and B cells, and some other nucleated cells that illicit acute immune rejection. The binding of prednisolone to glucocorticoid receptors on transplanted cells inhibits the downstream nuclear factor-*κ*В mediated expression of growth factors and secretion of proinflammatory cytokines (122). The binding inhibits the proliferation of several leukocytes including T and B cells, monocytes, macrophages and granulocyte, and made steroids a standard immunosuppressant for heart transplant recipients for induction and maintenance of immunosuppressive state in recipient patients. Long-term consumption of glucocorticoids is also coupled with undesired side effects on the heart as well as other organs (123).

#### 6.1.3. Antibodies

Abatacept is a human cytotoxic T-lymphocyte antigen (CTLA4)-Ig fusion protein which was initially developed as an inhibitor of the CD28/B7 pathway (BMS-188667) (124). The antibody is used to block the interaction of CD80 and CD86 of the antigen-presenting cells with CD28 costimulatory molecule on T cells to prevent its activation. In primates, CTLA4-Ig was tested and found to be effective in suppressing the acute rejection of MHC-mismatched renal allografts (125) and preventing the antibody formation against ovine red blood cells (126) in cynomolgus monkeys.

Basiliximab and daclizumab are chimeric human/murine monoclonal antibodies both targeting the alpha chain of CD25 high-affinity interleukin2 receptor of T cells, and preventing activation of T cells (127). The monoclonal antibody is largely used in renal transplantation with a significantly low acute rejection rate, which also allows rooms to lower the dosage of calcineurin inhibitors or steroids postoperatively.

#### 6.1.4. Mycophenolate mofetil

Mycophenolate mofetil (MMF, or RS-61443), and its hydrolyzed active form mycophenolic acid, inhibits inosine-monophosphate-dehydrogenase isoenzymes I and II, the rate-limiting enzymes crucial in *de novo* guanosine nucleotide synthesis (128). The inhibition of purine synthesis impedes the proliferation of stimulated T-lymphocytes, as well as the vessel cells such as smooth muscle cells (129), fibroblasts (130) and endothelial cells (131). Unlike other immunosuppressants, MMF can also reduce the prevalence of vascular graft disease, one of the main causes of allograft failure due to the progressive development of intimal hyperplasia. In a swine study, MMF also showed to abrogate cardiac allograft vasculopathy and increased graft survival (132). MMF is known to cause gastrointestinal intolerance or toxicity and this serves as the basis of the MMF dose reduction in patients who underwent allotransplantation (133). Studies have also shown that MMF dose reduction also increased the incidence of sustained rejection (134).

#### 6.1.5. Multiple drug regimen

In most of the cardiomyocyte transplantation studies using large animals, immunosuppression was achieved with multiple drug regimens (MDR) which combine multiple types of immunosuppressants. In a study by Zhu et al. (2018)., the group tested cardiovascular progenitor cell (CVPC) transplantation into an MI cynomolgus monkey model (28). In their study, they found that cyclosporine (30–45 mg/kg) alone could not effectively reduce immune rejection of CVPCs. This outcome was greatly improved by delivery of a multiple-drug regimen (MDR) consisting of cyclosporine (30–45 mg/kg/day), methylprednisolone (1 mg/kg/day with a loading dose of 500 mg), and basiliximab (1.5 mg/kg/day from day 1 till day 4), evident by the presence of the transplanted cells after 28 days of transplant. Yet, the transplanted cells were not detected after 140 days. On the other hand, Murry's laboratory used cyclosporin, methylprednisolone and abatacept in their human cardiomyocytes-to-macaque heart studies (12, 20, 22). They reported no evidence of all rejection with their MDR. In line with the finding, Romagnuolo and colleagues (2019) who employed the same MDR combination also showed minimal cellular rejection based on the grading criteria for human heart allografts (13).

Some interesting modifications in Murry's MDR were noticed comparing their two macaque studies and one study in swine, particularly the dosage of methylprednisolone. In Chong et al. (2014), methylprednisolone was given at 500 mg on the day of cell administration, and the dosage was maintained at 0.1–1.5 mg/kg until the animals were sacrificed. They did not observe any graft rejection. However in Liu's study (2017), methylprednisolone was reduced to 30 mg/kg on the day of cell delivery, and the maintenance dose was adjusted/increased to 6 mg/kg for the subsequent 2 days and 3 mg/kg until the animals were sacrificed. One graft rejection was observed due to interrupted immunosuppression as a result of a damaged intravenous catheter. In their pig study, the dosage of methylprednisolone was, once again, adjusted to 3.0 mg/kg 2 days before transplantation until 2 weeks, down to 1.5 mg/kg for subsequent maintenance. The dosage of methylprednisolone was further reduced to 1.0 mg/kg in some experimental subjects due to complications by porcine cytomegalovirus and pneumocystis pneumonia. The reason behind these substantial changes in methylprednisolone from 2014 was not mentioned, despite the consistent dosage of the other two immunosuppressants Abatacept and cyclosporin used in their macaques' studies. CsA, however, was increased to achieve a serum trough level >400 ng/ml in their recent pig study (22).

## 7. Discussion

This review provides a comprehensive overview of the animal models for cardiac remuscularization study. Successful establishment of the model would need to be confirmed using multiple analyses and imaging such as echocardiography, magnetic resonance imaging, cardiac pressure-volume loop analysis etc. The choice of anaesthesia, analgesia and antibiotic regimen post-surgery is key to increasing the survival of the animal subjects carrying the injured hearts. Nevertheless, the method of choice is based on the experimental needs and objectives. Transplantation of allogeneic cells would require effective immunosuppression to avoid host-vs.-graft rejection of the cells. While the best regimen has not been concluded, the selection of the immunosuppressive strategy is generally aimed toward achieving low toxicity-related side effects, highly efficient immunosuppression, and a high rate of engraftment and survival of the transplanted cells.

Nonetheless, ongoing concerns regarding the incidence of arrhythmias post-CM transplantation (herein refers to engraftment arrhythmias), were possible due to the presence of the nodal cells within the transplanted cardiomyocytes (12, 13, 30, 135). This problem has become the primary impediment to advancing the therapy to clinical trials. Intensive research has been ongoing to decipher pathways that direct chamber-specific cardiomyocyte differentiation to eliminate the presence of nodal cells in the culture and increase the population of ventricular cardiomyocytes in the subsequently transplanted graft. Alternatively, using a pharmacological approach to mitigate engraftment arrhythmias could also be a viable option (22).

In 2014, the European Society of Cardiology Cellular Biology of the Heart Working Group issued a position paper to urge for improving the preclinical assessment of novel cardioprotective therapies. In the statement, the experts attributed the low translatability of laboratory findings into clinics to the lack of rigorous tests during the preclinical animal study (136). One of the shortcomings was the preference over using reductionist cell or rodent models than employing a more integrative large mammal I/R model which simulates clinical reality. We summarize the methodology from the most recent cardiac remuscularization studies using large animals to provide an overview of the differences in reporting between laboratories, and their strategies in establishing MI models, cell source and delivery, as well as post-operative care analgesia and immunosuppression regimen. To increase the reproducibility and transparency of any future *in vivo* work, adherent to the Animal Research: Reporting In Vivo Experiments (ARRIVE) guideline is urged to facilitate the minimum information and standard required to be included in reporting and publishing animal research experiments (137).

## Author contributions

YY, SKT, ZG and JJT conceived the review outline. YY, FFR and JJT wrote the manuscript. YKY, BS, ZG and JJT reviewed and revised the manuscript. All authors contributed to the article and approved the submitted version.
